# Supplementary material for: Genomic Investigation and Successful Containment of an Intermittent Common Source Outbreak of OXA-48-Producing Enterobacter cloacae Related to Hospital Shower Drains
Source: Microbiol Spectr. 2021 Nov 24;9(3):e01380-21. doi: 10.1128/Spectrum.01380-21 (PMC8612159; doi:10.1128/Spectrum.01380-21)

Supplementary Appendix

Genomic investigation and successful containment of an intermittent common source outbreak of OXA-48-producing *Enterobacter cloacae* related to hospital shower drains.

Content:

Table S1. Overview of Infection prevention and control measures to contain the outbreak and to eliminate environmental reservoirs of OXA-48 producing *Enterobacter cloacae*. 2

Table S2. Sequencing Statistics.. 3

Supplementary Figure S1. Overview of antimicrobial resistance genes in the study samples................ 7

Supplementary Figure S2. Root to tip analysis of the time-calibrated phylogeny. 8

Supplementary Figure S3. Tolerance of Enterobacter cloacae to 25% acetic acid and active oxygen ... 9

Table S1. Overview of Infection prevention and control measures to contain the outbreak and to eliminate environmental reservoirs of OXA-48 producing *Enterobacter cloacae*.

Target	Infection prevention and control measures
**Patient**	• Admission screening (rectal swab for CRE)<br>• Weekly screening (rectal and wound swab for CRE) during hospital stay<br>• Information flyer for all patients and visitors about IPC relevance and measures
**Environment**	• Environmental sampling of contaminated shower sinks<br>• Environmental sampling (other than showers sinks, in regular intervals): soaps, water drain in unclean workrooms, water drain in cleaning rooms, water drain in kitchens + sideboard/station kitchen, water drain in clean workrooms, siphon washbasin in patient's bathroom, washbasin water in patient bathroom, medical equipment (ECG, sono equipment etc), clean work surfaces, work surfaces unclean, door handles, toilet seats, rounds trolley, training bikes<br>• Eradication attempts of "positive" sinks using 25% acetic acid, 3x weekly<br>• Suspension of shower use, whenever possible.<br>• Water-free washing as shower alternative<br>• Weekly change of shower drain cover screens and reprocessing/decontamination<br>• Constructional interventions: visitor toilet converted into a second unclean workroom, installation of custom-made cover grids for the shower sinks
**Health-care workers**	• Training of health care workers (physicians and nurses)<br>• Improvement of basic hygiene by implementing an automated 24-h hand disinfectant consumption monitoring.<br>• Regular monitoring of adherence to hand hygiene by bioburden measurement with feedback (cite)<br>• process monitoring of CVC installation<br>• Regular update meetings involving the local health authority

Abbreviations: CRE=carbapenem-resistant Enterobacterales; IPC=infection prevention and control; ECG=echocardiograph; CVC=central venous catheter

Table S2. Sequencing Statistics

ID	mlst	pattern	Accession	coverage	#contigs	Largest contig	Total length	N50	N75	L50	L75
BK24297	66	dnaA(52);fusA(21);gyrB(20);leuS(44);pyrG(45);rplB(4);rpoB(6)	SAMN12349713	49	46	932770	5006296	486681	225233	4	8
BK29742	66	dnaA(52);fusA(21);gyrB(20);leuS(44);pyrG(45);rplB(4);rpoB(6)	SAMN19988615	31	51	729970	4972596	383026	169273	5	9
BK30314	66	dnaA(52);fusA(21);gyrB(20);leuS(44);pyrG(45);rplB(4);rpoB(6)	SAMN19988616	36	45	1032931	5006792	434691	225888	4	8
D1389	66	dnaA(52);fusA(21);gyrB(20);leuS(44);pyrG(45);rplB(4);rpoB(6)	SAMN19988617	45	54	730254	5005444	374725	225233	5	9
D1970	66	dnaA(52);fusA(21);gyrB(20);leuS(44);pyrG(45);rplB(4);rpoB(6)	SAMN19988618	27	45	791110	4984828	427693	225888	5	8
D1999	66	dnaA(52);fusA(21);gyrB(20);leuS(44);pyrG(45);rplB(4);rpoB(6)	SAMN19988619	160	82	354436	4995168	149657	103194	12	22
D2026	66	dnaA(52);fusA(21);gyrB(20);leuS(44);pyrG(45);rplB(4);rpoB(6)	SAMN19988620	39	54	770460	5102431	486663	121101	4	11
D2170	66	dnaA(52);fusA(21);gyrB(20);leuS(44);pyrG(45);rplB(4);rpoB(6)	SAMN19988621	49	52	937730	4987940	372123	173312	5	9
D2223	66	dnaA(52);fusA(21);gyrB(20);leuS(44);pyrG(45);rplB(4);rpoB(6)	SAMN19988622	49	49	622370	5007779	311144	167379	6	11
D2224	66	dnaA(52);fusA(21);gyrB(20);leuS(44);pyrG(45);rplB(4);rpoB(6)	SAMN19988623	46	44	721504	4998000	366396	167379	5	10
D2226	66	dnaA(52);fusA(21);gyrB(20);leuS(44);pyrG(45);rplB(4);rpoB(6)	SAMN19988624	52	50	747710	4763648	486663	154005	4	8
D2230	66	dnaA(52);fusA(21);gyrB(20);leuS(44);pyrG(45);rplB(4);rpoB(6)	SAMN19988625	44	49	748491	5003219	374725	284185	5	9
D2242	66	dnaA(52);fusA(21);gyrB(20);leuS(44);pyrG(45);rplB(4);rpoB(6)	SAMN19988626	42	57	525937	4969120	223539	119482	7	15
D2243	66	dnaA(52);fusA(21);gyrB(20);leuS(44);pyrG(45);rplB(4);rpoB(6)	SAMN19988627	42	53	640666	5000016	284186	158486	6	12
D2249	66	dnaA(52);fusA(21);gyrB(20);leuS(44);pyrG(45);rplB(4);rpoB(6)	SAMN19988628	100	50	748491	4989673	311144	161152	6	11
D2250	66	dnaA(52);fusA(21);gyrB(20);leuS(44);pyrG(45);rplB(4);rpoB(6)	SAMN19988629	47	55	744006	4962233	374725	148320	5	10
D2251	66	dnaA(52);fusA(21);gyrB(20);leuS(44);pyrG(45);rplB(4);rpoB(6)	SAMN19988630	48	46	1084084	4908480	486681	225233	4	7
D2252	66	dnaA(52);fusA(21);gyrB(20);leuS(44);pyrG(45);rplB(4);rpoB(6)	SAMN19988631	41	55	748491	4964808	486663	148320	4	9
D2253	66	dnaA(52);fusA(21);gyrB(20);leuS(44);pyrG(45);rplB(4);rpoB(6)	SAMN19988632	46	48	748491	4991215	331499	155445	5	9
D2264	66	dnaA(52);fusA(21);gyrB(20);leuS(44);pyrG(45);rplB(4);rpoB(6)	SAMN19988633	53	43	1032931	4987959	486681	225233	4	7
D2361	66	dnaA(52);fusA(21);gyrB(20);leuS(44);pyrG(45);rplB(4);rpoB(6)	SAMN19988634	52	57	721625	5014364	368183	148320	5	10
D2362	66	dnaA(52);fusA(21);gyrB(20);leuS(44);pyrG(45);rplB(4);rpoB(6)	SAMN19988635	46	54	721483	4997799	368183	148320	5	10
D2363	66	dnaA(52);fusA(21);gyrB(20);leuS(44);pyrG(45);rplB(4);rpoB(6)	SAMN19988636	45	48	1032932	4986763	383026	169273	4	8
D2364	66	dnaA(52);fusA(21);gyrB(20);leuS(44);pyrG(45);rplB(4);rpoB(6)	SAMN19988637	40	60	743465	5036666	486663	130099	4	11
D2365	66	dnaA(52);fusA(21);gyrB(20);leuS(44);pyrG(45);rplB(4);rpoB(6)	SAMN19988638	49	46	747568	4984011	383026	282283	5	9
D2366	66	dnaA(52);fusA(21);gyrB(20);leuS(44);pyrG(45);rplB(4);rpoB(6)	SAMN19988639	44	49	747865	4980520	486681	153188	4	9
D2367	66	dnaA(52);fusA(21);gyrB(20);leuS(44);pyrG(45);rplB(4);rpoB(6)	SAMN19988640	40	56	741348	4939470	311144	149276	5	10
D2368	66	dnaA(52);fusA(21);gyrB(20);leuS(44);pyrG(45);rplB(4);rpoB(6)	SAMN19988641	43	53	709734	4961716	383026	299841	5	9
D2369	66	dnaA(52);fusA(21);gyrB(20);leuS(44);pyrG(45);rplB(4);rpoB(6)	SAMN19988642	43	68	625698	4995302	311144	118819	6	14
D2370	66	dnaA(52);fusA(21);gyrB(20);leuS(44);pyrG(45);rplB(4);rpoB(6)	SAMN19988643	46	54	721483	4969187	311144	148320	5	10
D2371	66	dnaA(52);fusA(21);gyrB(20);leuS(44);pyrG(45);rplB(4);rpoB(6)	SAMN19988644	46	53	721502	4981609	368183	148320	5	10
D2902	127	dnaA(46);fusA(20);gyrB(74);leuS(44);pyrG(45);rplB(24);rpoB(6)	SAMN19988645	64	44	851728	4759528	302960	149368	6	11
D2980	66	dnaA(52);fusA(21);gyrB(20);leuS(44);pyrG(45);rplB(4);rpoB(6)	SAMN19988646	35	49	748491	4983001	486663	139605	4	9
D2981	66	dnaA(52);fusA(21);gyrB(20);leuS(44);pyrG(45);rplB(4);rpoB(6)	SAMN19988647	84	55	722130	4998353	311144	139605	5	11
D2982	66	dnaA(52);fusA(21);gyrB(20);leuS(44);pyrG(45);rplB(4);rpoB(6)	SAMN19988648	39	50	748491	4998753	486663	158487	4	9
D2983	66	dnaA(52);fusA(21);gyrB(20);leuS(44);pyrG(45);rplB(4);rpoB(6)	SAMN19988649	39	50	742209	4988325	374026	169273	5	9
D2984	66	dnaA(52);fusA(21);gyrB(20);leuS(44);pyrG(45);rplB(4);rpoB(6)	SAMN19988650	44	51	1032429	4999500	486741	148320	4	9
D3070	66	dnaA(52);fusA(21);gyrB(20);leuS(44);pyrG(45);rplB(4);rpoB(6)	SAMN19988651	44	52	731817	5005484	372768	225233	5	9
D3078	66	dnaA(52);fusA(21);gyrB(20);leuS(44);pyrG(45);rplB(4);rpoB(6)	SAMN19988652	54	51	1033998	4982470	486828	148320	4	8

D3081	66	dnaA(52);fusA(21);gyrB(20);leuS(44);pyrG(45);rplB(4);rpoB(6)	SAMN19988653	85	56	1032910	5000735	383026	148320	5	9
D3082	66	dnaA(52);fusA(21);gyrB(20);leuS(44);pyrG(45);rplB(4);rpoB(6)	SAMN19988654	62	53	746989	4981142	486663	153188	4	9
D3083	66	dnaA(52);fusA(21);gyrB(20);leuS(44);pyrG(45);rplB(4);rpoB(6)	SAMN19988655	58	53	747985	4982442	486663	228343	4	8
D3084	66	dnaA(52);fusA(21);gyrB(20);leuS(44);pyrG(45);rplB(4);rpoB(6)	SAMN19988656	59	53	740416	4983938	486681	158487	4	9
D3085	66	dnaA(52);fusA(21);gyrB(20);leuS(44);pyrG(45);rplB(4);rpoB(6)	SAMN19988657	69	57	748491	4999292	486663	236877	4	8
D3086	66	dnaA(52);fusA(21);gyrB(20);leuS(44);pyrG(45);rplB(4);rpoB(6)	SAMN19988658	76	56	748490	5002793	311144	158487	5	10
D3087	66	dnaA(52);fusA(21);gyrB(20);leuS(44);pyrG(45);rplB(4);rpoB(6)	SAMN19988659	57	53	748490	5002108	374725	284186	5	9
D3088	66	dnaA(52);fusA(21);gyrB(20);leuS(44);pyrG(45);rplB(4);rpoB(6)	SAMN19988660	41	41	747865	4841902	374725	228233	5	8
D3089	-	dnaA(52);fusA(21);gyrB(20);leuS(44);pyrG(-);rplB(4);rpoB(6)	SAMN19988661	38	40	748491	4800011	374725	228343	5	9
D3090	66	dnaA(52);fusA(21);gyrB(20);leuS(44);pyrG(45);rplB(4);rpoB(6)	SAMN19988662	46	58	748491	4954153	374725	193010	5	9
D3091	66	dnaA(52);fusA(21);gyrB(20);leuS(44);pyrG(45);rplB(4);rpoB(6)	SAMN19988663	34	59	790156	4953093	374026	142626	5	10
D3092	-	dnaA(52);fusA(21);gyrB(20);leuS(44);pyrG(-);rplB(4);rpoB(6)	SAMN19988664	41	55	721625	4949120	486663	149950	4	9
D3093	-	dnaA(52);fusA(21);gyrB(20);leuS(44);pyrG(-);rplB(4);rpoB(6)	SAMN19988665	39	56	721504	4939587	486681	148320	4	9
D951	66	dnaA(52);fusA(21);gyrB(20);leuS(44);pyrG(45);rplB(4);rpoB(6)	SAMN19988666	27	44	1032932	4992100	383026	225233	4	8
D952	66	dnaA(52);fusA(21);gyrB(20);leuS(44);pyrG(45);rplB(4);rpoB(6)	SAMN19988667	45	44	722458	5007102	383024	225233	5	9
D965	66	dnaA(52);fusA(21);gyrB(20);leuS(44);pyrG(45);rplB(4);rpoB(6)	SAMN19988668	24	34	1016884	4846430	486663	223984	4	7
KE10000	96	dnaA(57);fusA(39);gyrB(49);leuS(57);pyrG(49);rplB(12);rpoB(20)	SAMN19988669	22	58	667504	5149019	269554	151351	6	13
KE10004	96	dnaA(57);fusA(39);gyrB(49);leuS(57);pyrG(49);rplB(12);rpoB(20)	SAMN19988670	30	48	943664	5114220	267329	151923	6	12
KE10011	96	dnaA(57);fusA(39);gyrB(49);leuS(57);pyrG(49);rplB(12);rpoB(20)	SAMN19988671	34	56	943666	5144974	269554	131255	5	12
KE10016	231	dnaA(46);fusA(20);gyrB(20);leuS(96);pyrG(45);rplB(29);rpoB(54)	SAMN19988672	56	56	677797	4968545	338735	213079	5	10
KE2473	66	dnaA(52);fusA(21);gyrB(20);leuS(44);pyrG(45);rplB(4);rpoB(6)	SAMN19988673	51	46	748491	4990948	486663	225888	4	8
KE2724	66	dnaA(52);fusA(21);gyrB(20);leuS(44);pyrG(45);rplB(4);rpoB(6)	SAMN19988674	51	48	747986	5005798	486663	225234	4	9
KE2874	66	dnaA(52);fusA(21);gyrB(20);leuS(44);pyrG(45);rplB(4);rpoB(6)	SAMN19988675	48	45	1032911	5006740	486663	225888	4	7
KE2967	66	dnaA(52);fusA(21);gyrB(20);leuS(44);pyrG(45);rplB(4);rpoB(6)	SAMN12349698	126	51	621827	4981711	292254	130000	6	13
KE3085	66	dnaA(52);fusA(21);gyrB(20);leuS(44);pyrG(45);rplB(4);rpoB(6)	SAMN19988676	47	49	722130	4958313	374725	139605	5	10
KE3086	66	dnaA(52);fusA(21);gyrB(20);leuS(44);pyrG(45);rplB(4);rpoB(6)	SAMN19988677	53	44	1032931	5003155	486663	225233	4	7
KE3435	66	dnaA(52);fusA(21);gyrB(20);leuS(44);pyrG(45);rplB(4);rpoB(6)	SAMN19988678	49	43	1032931	4987931	441250	225888	4	8
KE3512	66	dnaA(52);fusA(21);gyrB(20);leuS(44);pyrG(45);rplB(4);rpoB(6)	SAMN19988679	48	53	726275	5009332	310510	198777	6	11
KE3525	66	dnaA(52);fusA(21);gyrB(20);leuS(44);pyrG(45);rplB(4);rpoB(6)	SAMN12349715	49	47	1032929	5006459	401786	223982	4	9
KE3599	-	dnaA(52);fusA(21);gyrB(20);leuS(44);pyrG(-);rplB(4);rpoB(6)	SAMN19988680	54	50	884077	4961600	372123	225234	5	9
KE4065	66	dnaA(52);fusA(21);gyrB(20);leuS(44);pyrG(45);rplB(4);rpoB(6)	SAMN19988681	46	46	984218	5004471	486663	225233	4	8
KE4068	66	dnaA(52);fusA(21);gyrB(20);leuS(44);pyrG(45);rplB(4);rpoB(6)	SAMN19988682	42	49	747865	4998498	486663	225234	4	8
KE4215	66	dnaA(52);fusA(21);gyrB(20);leuS(44);pyrG(45);rplB(4);rpoB(6)	SAMN19988683	52	47	721483	5001913	372123	169273	5	9
KE8308	66	dnaA(52);fusA(21);gyrB(20);leuS(44);pyrG(45);rplB(4);rpoB(6)	SAMN19988684	40	53	729919	4991212	374725	173312	5	10
KE8695	66	dnaA(52);fusA(21);gyrB(20);leuS(44);pyrG(45);rplB(4);rpoB(6)	SAMN19988685	43	51	1032931	5005415	434691	154538	4	9
KE9025	66	dnaA(52);fusA(21);gyrB(20);leuS(44);pyrG(45);rplB(4);rpoB(6)	SAMN19988686	49	55	721504	5003353	272750	139605	7	13
KE9338	66	dnaA(52);fusA(21);gyrB(20);leuS(44);pyrG(45);rplB(4);rpoB(6)	SAMN19988687	47	50	747985	4988029	374725	169273	5	9
KE9354	66	dnaA(52);fusA(21);gyrB(20);leuS(44);pyrG(45);rplB(4);rpoB(6)	SAMN19988688	66	49	1033558	5005931	486663	225234	4	7
KE9355	-	dnaA(52);fusA(21);gyrB(20);leuS(44);pyrG(-);rplB(4);rpoB(6)	SAMN19988689	109	50	886315	4949960	486681	154538	4	8
KE9382	66	dnaA(52);fusA(21);gyrB(20);leuS(44);pyrG(45);rplB(4);rpoB(6)	SAMN19988690	27	49	722130	4983602	374725	152357	5	10
KE9428	66	dnaA(52);fusA(21);gyrB(20);leuS(44);pyrG(45);rplB(4);rpoB(6)	SAMN19988691	36	44	1021316	4968604	486663	225234	4	7
KE9468	66	dnaA(52);fusA(21);gyrB(20);leuS(44);pyrG(45);rplB(4);rpoB(6)	SAMN19988692	80	49	747589	4987545	383026	154538	5	9
KE9469	66	dnaA(52);fusA(21);gyrB(20);leuS(44);pyrG(45);rplB(4);rpoB(6)	SAMN19988693	76	50	748491	5004445	374315	284186	5	9
KE9472	66	dnaA(52);fusA(21);gyrB(20);leuS(44);pyrG(45);rplB(4);rpoB(6)	SAMN19988694	97	48	1084084	4907915	382653	154538	4	9

KE9476	66	dnaA(52);fusA(21);gyrB(20);leuS(44);pyrG(45);rplB(4);rpoB(6)	SAMN19988695	60	53	955676	4991470	486663	169273	4	8
KE9493	90	dnaA(58);fusA(37);gyrB(4);leuS(6);pyrG(42);rplB(4);rpoB(25)	SAMN19988696	44	52	1207762	5151391	530933	205911	3	7
KE9499	66	dnaA(52);fusA(21);gyrB(20);leuS(44);pyrG(45);rplB(4);rpoB(6)	SAMN19988697	66	47	747589	4965461	486681	225233	4	8
KE9510	90	dnaA(58);fusA(37);gyrB(4);leuS(6);pyrG(42);rplB(4);rpoB(25)	SAMN19988698	31	52	1207762	5151907	491022	194923	4	8
KE9527	66	dnaA(52);fusA(21);gyrB(20);leuS(44);pyrG(45);rplB(4);rpoB(6)	SAMN19988699	26	50	729919	4943143	339153	215683	5	9
KE9546	66	dnaA(52);fusA(21);gyrB(20);leuS(44);pyrG(45);rplB(4);rpoB(6)	SAMN19988700	44	51	747986	4963220	486663	225234	4	8
KE9721	-	dnaA(51);fusA(4);gyrB(4);leuS(~6);pyrG(68);rplB(30);rpoB(36)	SAMN19988701	37	45	554260	5128155	337518	253995	6	10
KE9733	66	dnaA(52);fusA(21);gyrB(20);leuS(44);pyrG(45);rplB(4);rpoB(6)	SAMN19988702	37	55	722130	5003379	295331	139605	5	11
KE9787	66	dnaA(52);fusA(21);gyrB(20);leuS(44);pyrG(45);rplB(4);rpoB(6)	SAMN19988703	36	46	721504	4968424	311144	158487	5	10
KE9798	66	dnaA(52);fusA(21);gyrB(20);leuS(44);pyrG(45);rplB(4);rpoB(6)	SAMN19988704	39	55	722130	4986530	311144	154538	5	10
KE9800	96	dnaA(57);fusA(39);gyrB(49);leuS(57);pyrG(49);rplB(12);rpoB(20)	SAMN19988705	39	52	667844	5127184	277939	152570	6	13
KE9837	66	dnaA(52);fusA(21);gyrB(20);leuS(44);pyrG(45);rplB(4);rpoB(6)	SAMN19988706	36	47	1032932	4985861	383026	169273	4	8
KE9866	66	dnaA(52);fusA(21);gyrB(20);leuS(44);pyrG(45);rplB(4);rpoB(6)	SAMN19988707	42	45	984239	4988399	486681	225233	4	8
KE9880	66	dnaA(52);fusA(21);gyrB(20);leuS(44);pyrG(45);rplB(4);rpoB(6)	SAMN19988708	51	46	747844	4984934	486663	225888	4	8
KE9883	66	dnaA(52);fusA(21);gyrB(20);leuS(44);pyrG(45);rplB(4);rpoB(6)	SAMN19988709	35	50	622246	4991279	311144	221341	6	11
KE9887	66	dnaA(52);fusA(21);gyrB(20);leuS(44);pyrG(45);rplB(4);rpoB(6)	SAMN19988710	33	53	638383	4992085	311144	157081	5	11
KE9969	66	dnaA(52);fusA(21);gyrB(20);leuS(44);pyrG(45);rplB(4);rpoB(6)	SAMN19988711	52	50	747865	4970987	311144	158487	5	10
KE9970	96	dnaA(57);fusA(39);gyrB(49);leuS(57);pyrG(49);rplB(12);rpoB(20)	SAMN19988712	48	57	829754	5148776	269554	131255	5	12
KT521	66	dnaA(52);fusA(21);gyrB(20);leuS(44);pyrG(45);rplB(4);rpoB(6)	SAMN19988713	24	44	741369	4801121	311144	225233	5	9
KT522	66	dnaA(52);fusA(21);gyrB(20);leuS(44);pyrG(45);rplB(4);rpoB(6)	SAMN19988714	29	50	623336	4855780	373288	148320	5	10
KT523	66	dnaA(52);fusA(21);gyrB(20);leuS(44);pyrG(45);rplB(4);rpoB(6)	SAMN19988715	27	46	730127	4771772	372123	228346	5	9
KT529	66	dnaA(52);fusA(21);gyrB(20);leuS(44);pyrG(45);rplB(4);rpoB(6)	SAMN19988716	58	55	748215	5001019	486681	284186	4	8
KT530	66	dnaA(52);fusA(21);gyrB(20);leuS(44);pyrG(45);rplB(4);rpoB(6)	SAMN19988717	47.2058	46	729919	4955712	374725	154538	5	10
KT609	66	dnaA(52);fusA(21);gyrB(20);leuS(44);pyrG(45);rplB(4);rpoB(6)	SAMN19988718	19	51	730255	4950499	486741	142038	4	10
KT610	66	dnaA(52);fusA(21);gyrB(20);leuS(44);pyrG(45);rplB(4);rpoB(6)	SAMN19988719	39	44	747589	4973961	311144	158487	5	10
KT614	66	dnaA(52);fusA(21);gyrB(20);leuS(44);pyrG(45);rplB(4);rpoB(6)	SAMN19988720	33	44	1021437	4980012	486681	225234	4	7
KT615	66	dnaA(52);fusA(21);gyrB(20);leuS(44);pyrG(45);rplB(4);rpoB(6)	SAMN19988721	34	46	747710	4985717	383026	225233	5	9
KT616	66	dnaA(52);fusA(21);gyrB(20);leuS(44);pyrG(45);rplB(4);rpoB(6)	SAMN19988722	39	48	747864	4972448	374725	169273	5	10
KT617	66	dnaA(52);fusA(21);gyrB(20);leuS(44);pyrG(45);rplB(4);rpoB(6)	SAMN19988723	39	49	721625	4996824	366396	167681	5	10
KT618	66	dnaA(52);fusA(21);gyrB(20);leuS(44);pyrG(45);rplB(4);rpoB(6)	SAMN19988724	35	51	937730	4989115	486828	225234	4	8
KT627	66	dnaA(52);fusA(21);gyrB(20);leuS(44);pyrG(45);rplB(4);rpoB(6)	SAMN19988725	49	49	747986	4990151	374725	206930	5	9
KT657	66	dnaA(52);fusA(21);gyrB(20);leuS(44);pyrG(45);rplB(4);rpoB(6)	SAMN19988726	51	55	729919	4966890	486681	228344	4	8
KT702	66	dnaA(52);fusA(21);gyrB(20);leuS(44);pyrG(45);rplB(4);rpoB(6)	SAMN19988727	30	55	746868	4982824	486681	154538	4	9
KT750	66	dnaA(52);fusA(21);gyrB(20);leuS(44);pyrG(45);rplB(4);rpoB(6)	SAMN19988728	32	47	721483	4966807	311144	169273	5	9
KT823-1	66	dnaA(52);fusA(21);gyrB(20);leuS(44);pyrG(45);rplB(4);rpoB(6)	SAMN19988729	33	51	721483	4991546	374725	154538	5	9
KT823-2	66	dnaA(52);fusA(21);gyrB(20);leuS(44);pyrG(45);rplB(4);rpoB(6)	SAMN19988730	25	64	721670	4979928	311144	133169	6	12
KT823-3	-	dnaA(52);fusA(21);gyrB(20);leuS(44);pyrG(-);rplB(4);rpoB(6)	SAMN19988731	31	45	644023	4892212	374725	159045	5	10
KT823-4	66	dnaA(52);fusA(21);gyrB(20);leuS(44);pyrG(45);rplB(4);rpoB(6)	SAMN19988732	32	57	721504	5003906	318459	168461	5	10
KT823-5	66	dnaA(52);fusA(21);gyrB(20);leuS(44);pyrG(45);rplB(4);rpoB(6)	SAMN19988733	33	59	721625	5003235	318459	154538	5	10
KT826-1	66	dnaA(52);fusA(21);gyrB(20);leuS(44);pyrG(45);rplB(4);rpoB(6)	SAMN19988734	24	54	721625	4975961	318459	168461	5	10
KT826-2	66	dnaA(52);fusA(21);gyrB(20);leuS(44);pyrG(45);rplB(4);rpoB(6)	SAMN19988735	28	52	721670	4975585	287752	139605	6	12
KT826-4	66	dnaA(52);fusA(21);gyrB(20);leuS(44);pyrG(45);rplB(4);rpoB(6)	SAMN19988736	47	47	721504	4987969	383026	169273	5	9
KT826-5	66	dnaA(52);fusA(21);gyrB(20);leuS(44);pyrG(45);rplB(4);rpoB(6)	SAMN19988737	41	55	721625	5006235	318459	154538	5	10
KT827-1	66	dnaA(52);fusA(21);gyrB(20);leuS(44);pyrG(45);rplB(4);rpoB(6)	SAMN19988738	51	54	721483	5003876	318459	168461	5	10

KT827-2	66	dnaA(52);fusA(21);gyrB(20);leuS(44);pyrG(45);rplB(4);rpoB(6)	SAMN19988739	68	58	721483	5003115	318459	154538	5	10
KT827-3	66	dnaA(52);fusA(21);gyrB(20);leuS(44);pyrG(45);rplB(4);rpoB(6)	SAMN19988740	69	61	721504	5005024	318459	139605	5	11
KT827-4	66	dnaA(52);fusA(21);gyrB(20);leuS(44);pyrG(45);rplB(4);rpoB(6)	SAMN19988741	33	58	722130	5003609	318459	154538	5	10
KT827-5	66	dnaA(52);fusA(21);gyrB(20);leuS(44);pyrG(45);rplB(4);rpoB(6)	SAMN19988742	100	58	721504	4988163	318459	154538	5	10
KT828-1	66	dnaA(52);fusA(21);gyrB(20);leuS(44);pyrG(45);rplB(4);rpoB(6)	SAMN19988743	36	55	721504	5005829	318459	168479	5	10
KT828-2	66	dnaA(52);fusA(21);gyrB(20);leuS(44);pyrG(45);rplB(4);rpoB(6)	SAMN19988744	34	51	640674	4987966	374725	169273	5	10

Supplementary Figure S1. Overview of antimicrobial resistance genes in the study samples.

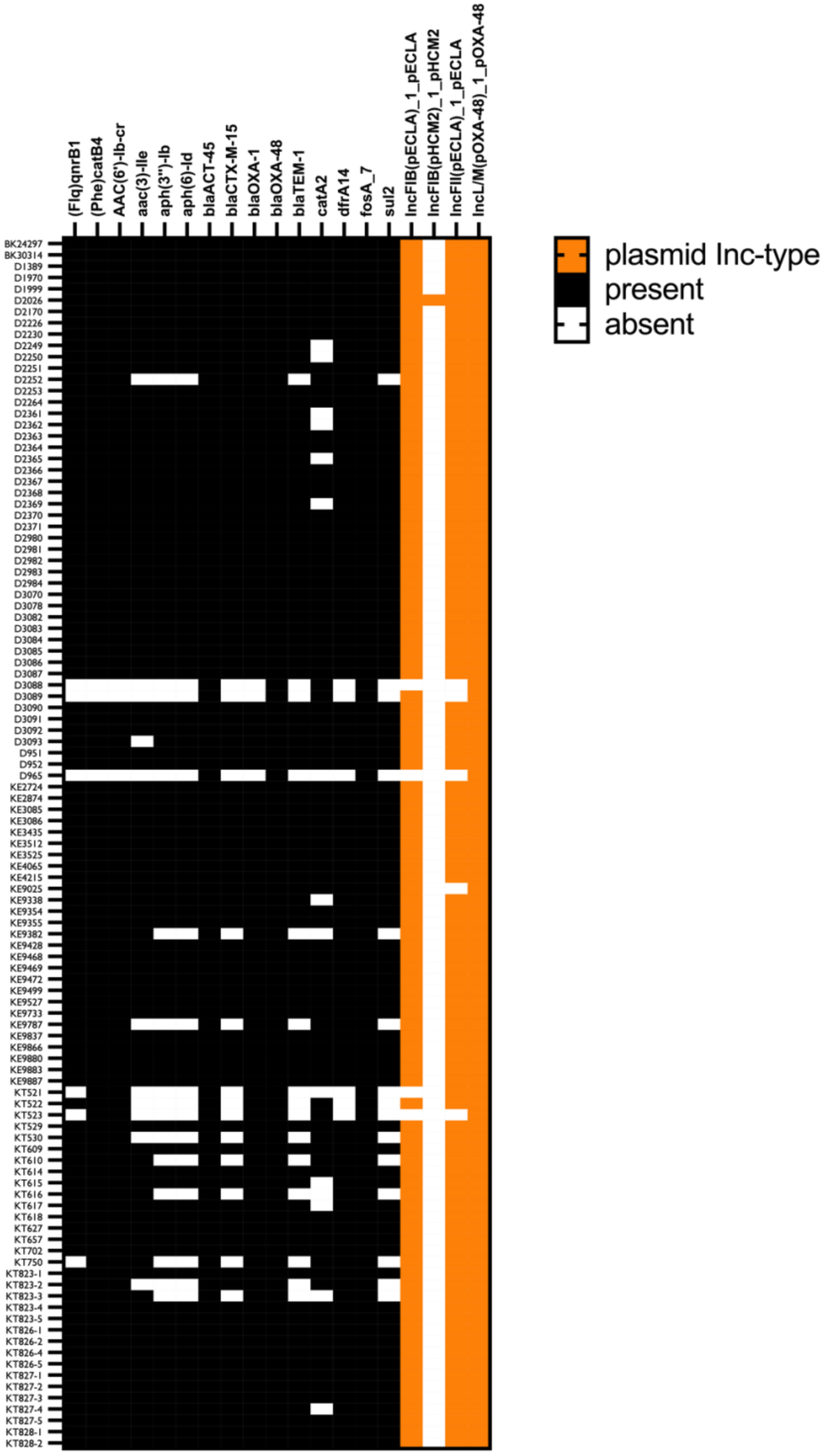

Supplementary Figure S2. Root to tip analysis of the time-calibrated phylogeny. The date of isolation of each isolate is color coded (from blue to red). A linear regression between the distance from root to tip with the date of isolation is performed to assess the influence of the temporal signal on the phylogenetic tree.

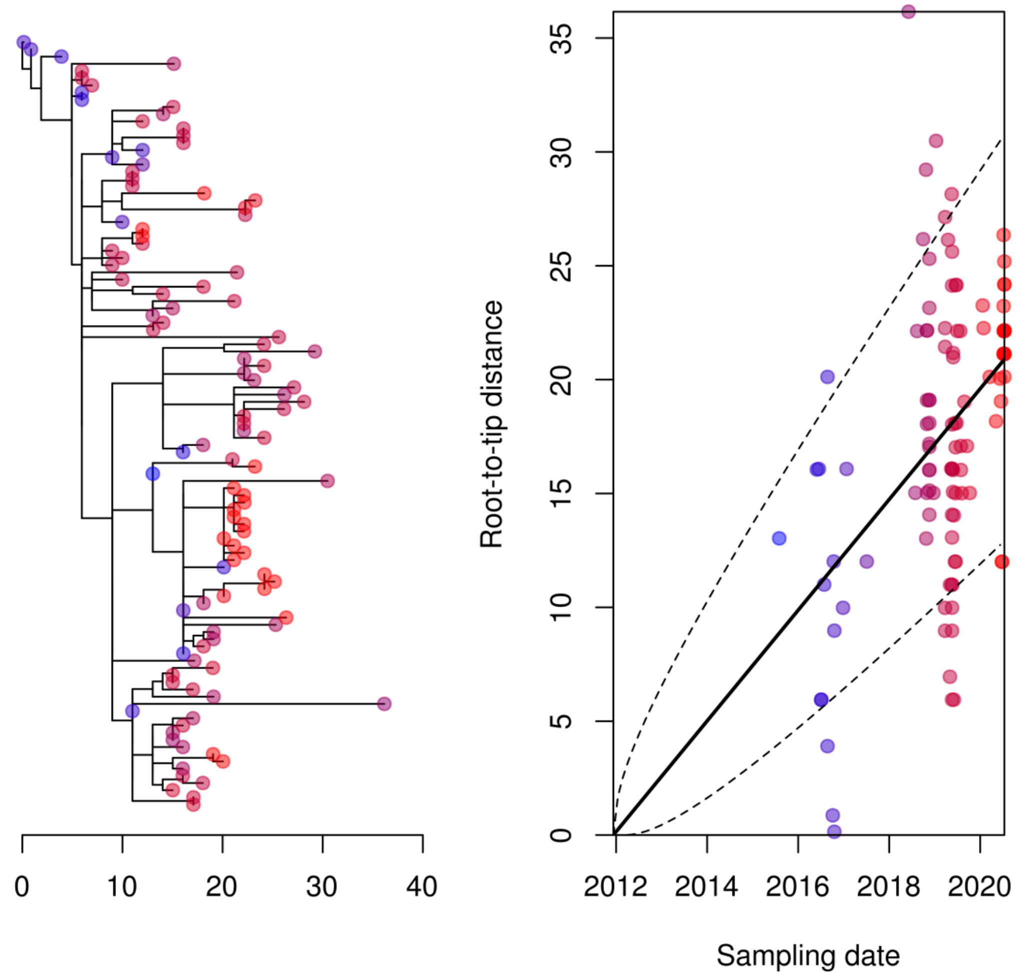

Supplementary Figure S3. Tolerance of Enterobacter cloacae to 25% acetic acid and active oxygen

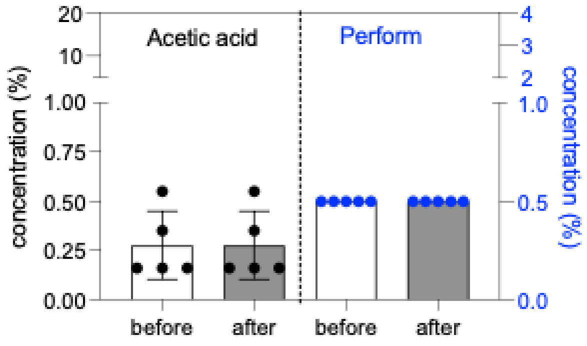

Supplement: SUPPLEMENTAL FILE 1 — Supplemental material. Download SPECTRUM01380-21_Supp_1_seq7.pdf, PDF file, 0.9 MB [file spectrum01380-21_supp_1_seq7.pdf]
